# Inequalities in cognitive impairment among older adults in China and the associated social determinants: a decomposition approach

**DOI:** 10.1186/s12939-021-01422-5

**Published:** 2021-03-19

**Authors:** Qingwen Deng, Wenbin Liu

**Affiliations:** School of Public Health, Fujian Medical University, Room 108 in the Building for School of Public Health, No. 1 Xuefubei Road, Minhou District, Fuzhou, 350122 China

**Keywords:** Income-related inequalities, Cognitive impairment, Older adults, Concentration index, Decomposition analysis, China

## Abstract

**Background:**

Despite there is growing evidence focusing on health inequalities in China, socioeconomic inequalities in cognitive impairment among older adults have received little attention. This study aims to measure socioeconomic inequalities in cognitive impairment among Chinese older adults, and determine the contributing social factors to the inequalities.

**Methods:**

A cross-sectional analysis was performed using data from the 2018 Chinese Longitudinal Healthy Longevity Survey (CLHLS). A total of 10,556 older adults aged 65 and over were included in the study. The prevalence of cognitive impairment was measured by using the Chinese version of the Mini-Mental State Examination. The socioeconomic inequalities in cognitive impairment were illustrated and quantified by the concentration curve and normalized concentration index. Multivariate logistic regression was conducted to identify the associated factors of cognitive impairment. And decomposition analysis was further applied to decompose the contribution of each determinant to the observed inequalities in cognitive impairment.

**Results:**

The study indicated that the prevalence of cognitive impairment among Chinese older adults was 18.95%. The overall concentration index for cognitive impairment was − 0.046, which suggested a higher concentration of cognitive impairment among socioeconomically disadvantaged older adults. The results showed the prevalence of cognitive impairment was associated with sex, age, marital status, education level, occupation, economic status, emotional support, financial support, living arrangement, and participation in informal activities. Decomposition results further revealed the contributions of the determinants to the inequalities in cognitive impairment. Specifically, age (131.61%), marital status (85.68%), emotional support (84.85%), education level (39.73%), occupation (21.24%), sex (17.25%), financial support (− 4.19%), economic status (1.02%), living arrangement (0.88%), and informal activities (0.30%) have varying degrees of contributions to the inequality in cognitive impairment.

**Conclusion:**

This study sheds light on the pro-rich inequality in cognitive impairment among older adults in China. It suggests that policymakers should pay more attention to older adults who are female, old-old, widowed, illiterate, economically disadvantaged, with no social support, and less socially involved. Also, more targeted interventions should be undertaken to improve the socioeconomic conditions of these vulnerable individuals and strengthen their ability to cope with the risk of cognitive impairment.

## Background

Cognitive impairment is characterized by declines in attention, memory, reasoning, intelligence and other mental functions [1]. Older adults with severe cognitive impairment can lead to dementia, which is still incurable [2]. Dementia also can make the older adults lose the ability to accomplish daily life and independent living, which has a profound adverse impact on older people’s health and successful aging, and subsequently places a heavy burden on families and healthcare systems [3, 4]. In recent years, the prevalence of cognitive impairment has been expected to dramatically increase with rapid aging, which is increasingly become a great public health concern on a global scale. For example, China has the largest number of older adults in the world, as well as the largest population of patients with dementia, with nearly 25% of the world’s dementia cases and an annual increase of more than 0.36 million [5]. Thus, there is an urgent need to concentrates efforts to improve cognitive function and prevent the progression of cognitive impairment to dementia among older adults at high risks.

With the increasing demand for care for older adults with dementia, cognitive impairment issues have aroused great attention, such as the diagnosis and management of cognitive impairment [6] and the care of patients with cognitive impairment [7]. However, the inequality in cognitive impairment is still poorly understood. Only a small number of existing studies focus on describing the status of inequalities in cognitive impairment [8, 9] or the relationship between proposed factors and inequality in cognitive impairment [10, 11]. For instance, a study has indicated that women and rural populations have unfavorable inequalities in cognitive impairment compared with men and urban populations [9], and some other studies have suggested that the inequalities in cognitive impairment were related to individual characteristics and socioeconomic context [10–12]. Although researchers have found that cognitive impairment is unequally distributed among socioeconomic groups, namely, people of disadvantaged socioeconomic status are at greater risk for cognitive impairment, these studies did not assess the degree of health inequalities or determine how much of it was explained by the proposed factors.

Regarding the influencing factors of inequalities in cognitive impairment, several studies have investigated the impact of socioeconomic factors on cognitive ability. It is generally highlighted that better cognitive performance was associated with higher socioeconomic status [10, 13]. Specifically, educational inequality can widen racial/ethnic/nativity disparities in dementia for older adults [14], and offspring education has a profoundly positive influence on later-life cognitive health from a family perspective [15].

In addition to socioeconomic factors, social support and social participation have also been documented in the literature on social determinants for the inequalities in cognitive impairment. Social support is defined as “the support accessible to an individual through social ties to other individuals, groups, and the larger community” [16]. It generally includes emotional support and instrumental support [17]. Emotional support often refers to contact and care from family members, friends, and people who regularly associate. Instrumental support refers to tangible support such as financial or material assistance [18, 19]. With the deepening of the research on the relationship between social support and health of older adults, there are increasing studies believe that living arrangement should be incorporated into the scope of social support [20, 21]. Older people living alone, in care facilities, or with family members are considered to have a significant impact on their health [22, 23]. Compared with the “passive/inward” nature of social support, social participation is more endowed with the connotation of “active/outward”, that is to say, it emphasizes that individuals take the initiative to form contact with the outside world. Specifically, social participation refers to a person engages in social or community activities that interact with others [24]. The activities involved can be formal (such as organized activities) or informal (such as group leisure activities) [25]. Unlike individual or family activities, social participation activities place more emphasis on interaction with the community [26]. In sum, a growing body of evidence illustrates the protective influence of higher socioeconomic status [13], social support [27], and social participation [28] on cognitive impairment in older adults.

Those mentioned above are all social determinants. The influence of social determinants on cognitive health is universal, persistent and cumulative, which can imperceptibly affect the development of cognitive impairment, and gradually shape the inequality in cognitive health. More specifically, socioeconomic status can reflect the risk of cognitive impairment in people’s old age through the upper and lower limits of their access to a variety of resources [10, 29]. Social support helps to reduce the likelihood of cognitive impairment among older adults through the fulfillment that certain risks (loneliness, poverty) are resisted [20]. Social participation contributes to preventing cognitive impairment by maintaining cognitive function through positive connections with the outside world [28]. Cognitive impairment and its inequality vary with social determinants.

Increasing the understanding of the inequalities in cognitive impairment among different socio-economic groups is of great value to researchers and policymakers in developing meaningful targeted interventions to promote equity. To the best of our knowledge, prior research on the social factors of cognitive impairment in older adults is relatively scattered [30]. Additionally, no studies have paid attention to the measurement and quantification of inequalities in cognitive impairment. These research gaps lead to a dearth of evidence on the impact of multidimensional social factors on inequalities in cognitive impairment. Therefore, this study aims to determine the degree of inequalities and integrate its social determinants of cognitive impairment among older adults in China. The findings are promising for bridging the gap in the literature about the inequality in cognitive impairment. It will help tailor strategies to reduce the inequalities of cognitive health, especially echoes the increasing highlight of health equity in China’s health systems and many other countries.

## Methods

### Data source

The data used in this study are from the Chinese Longitudinal Health Longevity Survey (CLHLS), which is a large population-based study conducted by the Centre for Healthy Aging and Family Studies at Peking University and the Chinese Center for Disease Control and Prevention. The CLHLS study was approved by the Ethics Committee of Peking University (IRB00001052–13074), and all respondents or their proxies provided written informed consent. The CLHLS sampled older adults aged 65 and over from 22 out of the 31 provinces of mainland China, the population in these provinces constitutes approximately 85% of the total population [31].

The CLHLS targeted community-dwelling and institutionalized older adults. Using multistage stratified cluster sampling, octogenarians and nonagenarians were randomly selected based on gender and residence place (i.e., living in the same city, county, village, or street) for a given centenarian. All information was obtained in participants’ homes through face-to-face interviews using internationally compatible questionnaires by trained investigators. Proxy (the spouse or other family member) was instead interviewed when the participants were unable to answer questions, while questions about the cognitive function can only be answered by the participants themselves. The data quality of CLHLS has been reported satisfactory by previous studies [31].

Data of the study were obtained from the CLHLS in 2018, which surveyed 15,874 older adults. To ensure the analysis effectively, the samples with missing values or answers of “don’t know” in any variables of interest would be excluded. Finally, a total of 10,665 respondents were included in this study.

### Measures

#### Explained variable: cognitive impairment

The cognitive impairment of the respondents was assessed by the Chinese version of the Mini-Mental State Examination (MMSE) adapted from the scale developed by Folstein and colleagues [32], which has been proven to be reliable and valid for elderly Chinese [33, 34]. MMSE tests 24 items from the five aspects of cognitive function: orientation, reaction, attention & calculation, recall, and language. The total score ranged from 0 to 30, and the higher score indicated better cognitive ability. Education-based MMSE cutoff points are widely used to screen for cognitive impairment in the elderly with low education [35]. As quite many respondents (48.29%) in this study had no formal education, we used education-based MMSE cutoff points to define those who with cognitive impairment: < 18, respondents with no formal schooling; < 21, respondents with 1 to 6 years of schooling; and < 25, respondents with more than 6 years of schooling [36, 37].

#### Explanatory variables

Explanatory variables of the study consisted of socioeconomic factors, social support, and social participation.

Socioeconomic factors included education level, occupation, and economic status. Respondents were asked “How many years did you attend school?”, according to the answers of 0 years, 1 ~ 6 years, and 7 years or more, education level was classified into three categories: no schooling (1), primary school (2), and middle school or more (3). The categories of occupation in the questionnaire included professional and technical personnel, governmental, institutional or managerial personnel, commercial, service or industrial worker, self-employed, agriculture, forestry, animal husbandry or fishery worker, house worker, and others. In this study, the occupation was recoded into non-white-collar (1) and white-collar (2). Economic status was assessed by asking “the per capita of household income in the last year?” and then divided into quintiles with quintile 1 (1) indicating the poorest and quintile 5 (5) indicating the richest.

Social support was measured by three elements: emotional support, financial support, and living arrangement. Emotional support was obtained by the question “The first person to whom you usually talk frequently in daily life (including spouse, son/daughter, son-/daughter-in-law, grandchildren and their spouse, other relatives, friends/neighbors, social workers/housekeeper, network friends, and nobody).” The answers were grouped into four categories: nobody (1), relatives/friends/neighbors and others (2), children (3), and spouse (4). Financial support was calculated by the answers to three questions: “How much did you receive from your son(s) or daughter(s)-in-law, last year?”, “How much did you receive from your daughter(s) or son(s)-in-law last year?”, and “How much did you receive from your grandchild (ren) last year?”. If the answers to the three questions add up to 0, we assumed the respondent had no financial support (1); otherwise, he/she had financial support (2). Living arrangement was categorized into lived alone (1), lived in an institution (2), and lived with household members (3).

Social participation included two aspects of organized activities and informal activities. The questions for organized activities and informal activities were “Do you take part in some social activities (organized) at present?”, “Do you take part in the following activities (e.g., square dance, series, interact with friends, play cards/mah-jongg) at present?”, respectively. And the options of two questions both included “almost every day”, “not daily, but once for a week”, “not weekly, but at least once for a month, “not monthly, but sometimes“, and “never“. If the respondents select “never”, we assumed the respondent had not participated in organized activities or informal activities (1); otherwise, he/she had participated in organized activities or informal activities (2).

#### Covariates

Covariates included demographic factors such as sex, age, marital status, and residential area. Sex was defined as male (1) and female (2). Age was obtained by self-reported and then divided into three groups: aged 65 ~ 74 years old (1), aged 75 ~ 84 years old (2), and aged 85 years or above (3). Marital status was categorized into two groups: separated/divorced/widowed/never married (1), married and living with a spouse (2). The residential area was defined as urban/town (1) and rural (2).

### Statistical analysis

The Stata 15.1 software was used for data analysis. A difference analysis was performed with the Chi-square test. As the dependent variable is dichotomous, binary logistic regression was used to estimate the effects of demographic factors, social support, and social participation on cognitive impairment among older adults. In addition, the concentration curve and concentration index (C) were used to reflect the income-related inequality in cognitive impairment. In this study, the distribution of cognitive impairment was examined by economic status quintiles. The C is defined as twice the area between the concentration curve and the line of equality. The concentration curve is obtained by plotting the cumulative percentage of cognitive impairment (Y-axis) against the cumulative percentage of the population ranked by economic status (X-axis). The C can be calculated using the following formula [38]:

$$ C=\frac{2}{\mu \times \mathit{\operatorname{cov}}\left(h,r\right)} $$

where *h* is the health outcome (cognitive impairment in this study), *μ* is the mean of *h*, and *r* denotes the fractional rank of individuals in the distribution used (economic status quintiles). The C ranges between − 1 and + 1, a value of zero represents absolute fairness and there exists no income-related inequality. When C is positive, suggesting that cognitive impairment is more concentrated among rich people (pro-poor). Conversely, if the C takes a negative value, indicating cognitive impairment is more concentrated among poor people (pro-rich). As the outcome variable in the study is binary, the bounds of C do not vary between − 1 and + 1. To correct this issue, we followed Wagstaff’s suggestion [39], normalizing the C by dividing the estimated C by 1 minus the mean (1 − *μ*).

Decomposition analysis was further performed to determine the contribution of each factor to the inequality, in which the contribution of each factor is the product of the sensitivity or elasticity of cognitive impairment with respect to that factor and its degree of inequality.

## Results

Table 1 outlines the demographic characteristics of the included respondents (group of non-cognitive impairment vs. group of cognitive impairment) by independent variables. A total of 10,556 adults aged 65 and over with an average age of 84.74 (standard deviation [SD] = 11.55) years were included in this study, of which 5833 (55.26%) were females, 5187 (49.14%) were 85 years or older. The majority of respondents were separated/divorced/widowed/never married (57.76%) and resided in urban/town areas (55.83%). The respondents with no school years nearly accounted for half of the respondents (48.29%). Most of the respondents had engaged in non-white-collar occupation (88.50%). The proportion of reporting lack of emotional support, financial support, and lived alone was 2.16, 28.17, and 15.42%, respectively. In addition, 14.08 and 62.45% of the respondents, respectively, had participated in organized activities and informal activities.

**Table 1:** Descriptive statistics of the study population of non-cognitive impairment vs. cognitive impairment.

Variable	Prevalence of non- cognitive impairment (*n* = 8556)		Prevalence of cognitive impairment (*n* = 2000)		*χ*^2^
	*n*	%	*n*	%	
**Demographic factors**					
**Sex**					162.049***
Male	4083	86.45	640	13.55	
Female	4473	76.68	1360	23.32	
**Age (years old)**					1696.464***
65 ~ 74	2390	98.43	38	1.57	
75 ~ 84	2788	94.80	153	5.20	
≥ 85	3378	65.12	1809	34.88	
**Marital status**					781.131***
Separated/divorced/widowed/never married	4386	71.94	1711	28.06	
Currently married and living with a spouse	4170	93.52	289	6.48	
**Residential area**					1.269
Urban/Town	4799	81.44	1094	18.56	
Rural	3757	80.57	906	19.43	
**Socioeconomic status**					
**Education level**					514.650***
No schooling	3675	72.10	1422	27.90	
Primary school	3103	89.19	376	10.81	
Middle school or more	1778	89.80	202	10.20	
**Occupation**					44.838***
non-white-collar	7486	80.13	1856	19.87	
white-collar	1070	88.14	144	11.86	
**Economic status (quintile)**					18.861**
1 (poorest)	1696	80.34	415	19.66	
2	1683	79.73	428	20.27	
3	1687	79.88	425	20.12	
4	1712	81.10	399	18.90	
5 (richest)	1778	84.23	333	15.77	
**Social support**					
**Emotional support**					933.919***
Nobody	122	53.51	106	46.49	
Relatives/friends/neighbors and others	1164	87.85	161	12.15	
Children	3666	70.83	1510	29.17	
Spouse	3604	94.17	223	5.83	
**Financial support**					24.983***
No	2320	78.01	654	21.99	
Yes	6236	82.25	1346	17.75	
**Living arrangement**					61.264***
Alone	1412	86.52	220	13.48	
In an institution	189	68.73	86	31.27	
With household member(s)	6955	80.41	1694	19.59	
**Social participation**					
**Organized activities**					1.066
No	7366	81.21	1704	18.79	
Yes	1190	80.08	296	19.92	
**Informal activities**					11.444**
No	3147	79.39	817	20.61	
Yes	5399	81.90	1193	18.10	

The overall prevalence of cognitive impairment among the study population was 18.95%, significantly lower than those who were females (23.32%), aged 85 years or older (34.88%), Separated/divorced/widowed/never married (28.06%), lived in an institution (31.27%) or lived with household member (19.59%), and received no education (27.90%). Cognitive impairment showed a general income gradient, with a lower prevalence was found among people with a higher economic status. More specifically, the richest (quintile 5) had the lowest prevalence of cognitive impairment (15.77%). The prevalence of cognitive impairment was higher among respondents who had no emotional support (46.49%), had no financial support (21.99%), and not participated in informal activities (20.61%). However, there was no significant difference in the prevalence of cognitive impairment between residential areas and participation in organized activities (*P* > 0.05).

The logistic regression results for cognitive impairment and its determinants are presented in Table 2. Except for residential areas and whether participated in organized activities (*P* > 0.05), all other factors were predicted to influence cognitive impairment significantly. Among demographic factors, being female (OR = 1.257, 95%CI = 1.101 to 1.434) was significantly associated with higher odds of cognitive impairment. Compared to the young-old (aged 65 ~ 74 years), the old-old (aged 75 years or above) were estimated with higher odds of cognitive impairment. Special strikingly, those aged 85 years or above (OR = 20.486, 95%CI = 14.505 to 28.935) were at an alarmingly high risk of cognitive impairment. Older adults who were married and living with a spouse (OR = 0.690, 95%CI = 0.521 to 0.913) were less likely to suffer from cognitive impairment than the separated/divorced/widowed/never-married older adults. As for the education level, compared to the respondents who did not attend school, the respondents who had a primary school (OR = 0.678, 95%CI = 0.585 to 0.787) can significantly lower the odds of cognitive impairment. Compared with non-white-collar jobs, white-collar jobs (OR = 0.738, 95%CI = 0.580 to 0.937) had a lower risk of cognitive impairment. In economic status, we found that higher income was related to the lower risks of cognitive impairment. For example, the richest (OR = 0.577, 95%CI = 0.475 to 0.701) had the lowest odds of cognitive impairment. Furthermore, of social support, having different sources of emotional support (from relatives/friends/neighbors and others [OR = 0.219, 95%CI = 0.154 to 0.311; from children [OR = 0.400, 95%CI = 0.293 to 0.546]; from spouse [OR = 0.242, 95%CI = 0.160 to 0.366]) and having financial support (OR = 0.712, 95%CI = 0.630 to 0.804) were correlated with the lower risks of cognitive impairment. Regarding living arrangement, compared to those who lived alone, the respondents who lived in an institution had the highest odds of cognitive impairment (OR = 3.374, 95%CI = 2.425 to 4.695), followed by living with household members (OR = 2.227, 95%CI = 1.855 to 2.658). In terms of social participation, respondents who did participate in informal activities (OR = 0.872, 95%CI = 0.776 to 0.980) were less likely to suffer from cognitive impairment than those who did not participate in informal activities.

**Table 2:** Multivariable logistic regression analysis results for cognitive impairment

Variable	*OR*	S.E.	z	*p*-value	95% *CI*	
					Low	High
**Demographic factors**						
**Sex**						
Male	–	–	–	–	–	–
Female	1.257	0.085	3.39	0.001	1.101	1.434
**Age (years)**						
65 ~ 74	–	–	–	–	–	–
75 ~ 84	2.986	0.557	5.87	< 0.001	2.072	4.303
≥ 85	20.486	3.609	17.14	< 0.001	14.505	28.935
**Marital status**						
Separated/divorced/widowed/never married	–	–	–	–	–	–
Currently married and living with a spouse	0.690	0.099	−2.59	0.009	0.521	0.913
**Residential area**						
Urban/Town	–	–	–	–	–	–
Rural	0.996	0.059	−0.07	0.946	0.887	1.119
**Socioeconomic status**						
**Education level**						
No schooling	–	–	–	–	–	–
Primary school	0.678	0.051	−5.13	< 0.001	0.585	0.787
Middle school or more	1.223	0.142	1.74	0.082	0.975	1.535
**Occupation**						
non-white-collar	–	–	–	–	–	–
white-collar	0.738	0.090	−2.49	0.013	0.580	0.937
**Economic status (quintile)**						
1 (poorest)	–	–	–	–	–	–
2	0.861	0.078	−1.65	0.099	0.722	1.028
3	0.812	0.075	−2.27	0.023	0.678	0.972
4	0.771	0.073	−2.74	0.006	0.640	0.929
5 (richest)	0.577	0.057	−5.55	< 0.001	0.475	0.701
**Social support**						
**Emotional support**						
Nobody	–	–	–	–	–	–
Relatives/friends/neighbors and others	0.219	0.039	−8.48	< 0.001	0.154	0.311
Children	0.400	0.063	−5.78	< 0.001	0.293	0.546
Spouse	0.242	0.051	−6.71	< 0.001	0.160	0.366
**Financial support**						
No	–	–	–	–	–	–
Yes	0.712	0.044	−5.45	< 0.001	0.630	0.804
**Living arrangement**						
Alone	–	–	–	–	–	–
In an institution	3.374	0.569	7.22	< 0.001	2.425	4.695
With household member(s)	2.227	0.201	8.87	< 0.001	1.866	2.658
**Social participation**						
**Organized activities**						
No	–	–	–	–	–	–
Yes	1.065	0.089	0.76	0.449	0.905	1.253
**Informal activities**						
No	–	–	–	–	–	–
Yes	0.872	0.050	−2.30	0.021	0.776	0.980

**Fig. 1:**
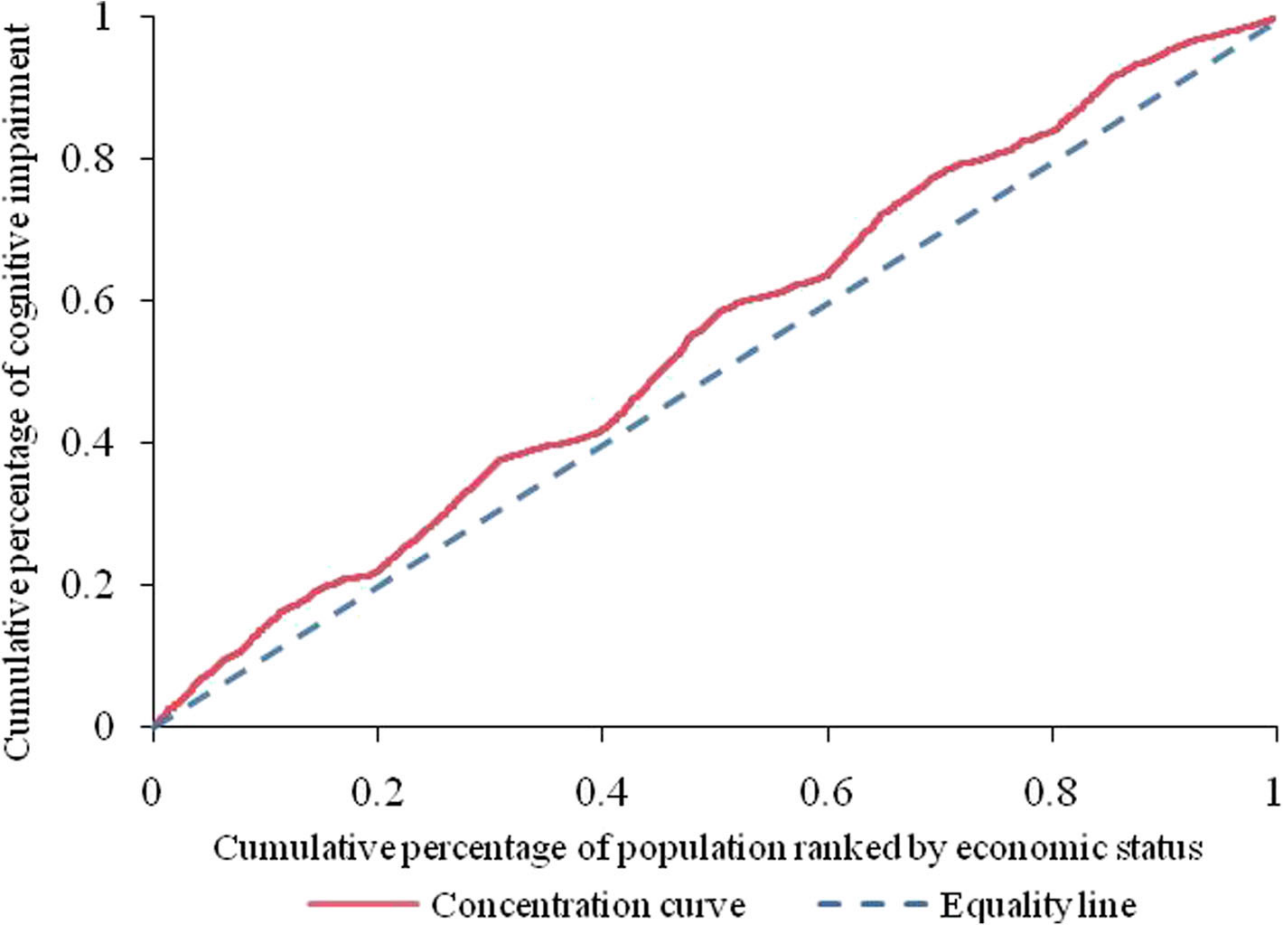
Concentration curve of cognitive impairment

The concentration curve of cognitive impairment among older adults is shown in Fig. 1. The curve lay above the equality line, with the negative value of the concentration index of − 0.046. These indicated that income-related health inequalities exist in the distribution of cognitive impairment. Furthermore, the inequality disadvantageous to the poor, namely, cognitive impairment was more concentrated among older people with lower economic status.

The results of the decomposition analysis of inequalities in cognitive impairment are illustrated in Table 3. Demographic factors such as age (133.61%), marital status (85.68%) and sex (17.25%) had the largest contribution to the pro-rich inequalities in cognitive impairment. Social support variables came next in the importance of the contribution, 84.85%, − 4.19, and 0.88% of inequalities in cognitive impairment can be explained by emotional support, financial support, and living arrangement, respectively. In third place were socioeconomic factors, with education level, occupation, and economic status accounting for − 39.73, 21.24%, and − 1.02% of inequalities in cognitive impairment. Social participation factors were fourth in importance, with informal activities (0.3%) making small contributions to the inequalities that disfavor the poor. Additionally, residential area (− 0.07%) and organized activities (0.18%) showed their insubstantial contributions to the inequalities. Furthermore, as indicated by the C, women, the old-old, separated/divorced/widowed/never married, resided in rural, with less education, had no emotional support, had financial support, lived in an institution, and not participated in informal activities were more concentrated among the poor, and they had higher probabilities of reporting the problem of cognitive impairment.

**Table 3:** Decomposition analysis of socioeconomic inequalities in cognitive impairment among Chinese older adults

Variable	Elasticity	C	Contribution to the C		
			Contribution	%	Summed percentage
**Demographic factors**					
**Sex**					17.25
Female (ref.)					
Male	−0.0918	0.0855	−0.0078	17.25	
**Age (years)**					133.61
≥ 85 (ref.)					
75 ~ 84	−0.4813	0.1100	−0.0529	116.36	
65 ~ 74	−0.6230	0.0126	− 0.0078	17.25	
**Marital status**					85.68
Currently married and living with a spouse (ref.)					
Separated/divorced/widowed/never married	0.1922	−0.2028	−0.0390	85.68	
**Residential area**					−0.07
Rural (ref.)					
Urban/Town	0.0020	0.0163	0.0000	−0.07	
**Socioeconomic status**					
**Education level**					−39.73
Middle school or more (ref.)					
Primary school	−0.1744	−0.0691	0.0120	−26.48	
No schooling	−0.0872	−0.0691	0.0060	−13.25	
**Occupation**					21.24
white-collar (ref.)					
non-white-collar	0.2416	−0.0400	−0.0097	21.24	
**Economic status (quintile)**					−1.02
5 (richest) (ref.)					
4	0.0521	0.0078	0.0004	−0.89	
3	0.0613	0.0017	0.0001	−0.23	
2	0.0719	−0.0002	0.0000	0.03	
1 (poorest)	0.0987	−0.0003	0.0000	0.07	
**Social support**					
**Emotional support**					84.85
Spouse (ref.)					
Children	0.2219	−0.1735	−0.0385	84.60	
Relatives/friends/neighbors and others	−0.0110	0.0093	−0.0001	0.23	
Nobody	0.0275	−0.0005	0.0000	0.03	
**Financial support**					−4.19
Yes (ref.)					
No	0.0859	0.0222	0.0019	−4.19	
**Living arrangement**					0.88
With household member(s) (ref.)					
In an institution	0.0097	−0.0539	−0.0005	1.15	
Alone	−0.1110	−0.0011	0.0001	−0.27	
**Social participation**					
**Organized activities**					0.18
Yes (ref.)					
No	−0.0486	0.0017	−0.0001	0.18	
**Informal activities**					0.30
Yes (ref.)					
No	0.0462	−0.0030	−0.0001	0.30	

## Discussion

To our knowledge, several studies have been conducted on the prevalence and risk factors affecting cognitive impairment among older adults, but there was a dearth of studies on the inequalities in cognitive impairment and its associated social determinants. This study quantified and decomposed income-related inequalities in cognitive impairment among older adults based on the national representative data from CLHLS. This approach can help us determine the income-related inequalities in cognitive impairment among older adults and the main causes, which will be vital for tailoring effective interventions to prevent or delay cognitive impairment among older adults.

The socioeconomic status of Chinese older adults can probably be inferred from the sample description. Nearly half of the samples (48.29%) did not attend school, and almost nine out of ten (88.50%) used to be engaged in the non-white-collar occupation. Due to the experience of wars and natural disasters at their school-age (about 1930s–1960s), few Chinese older adults have access to education. And lower education is usually associated with the non-white-collar occupation. In previous studies on older adults in China, the proportions without schooling are more than half, and non-white-collar workers are roughly around 90% [20, 40, 41]. In the 1950s, China’s rural population accounted for 89.36%, most of whom were farmers [42]. Those who were engaged in mechanical manual labor without schooling were generally in a lower socioeconomic status.

The results revealed that cognitive impairment among older adults in China is unequally distributed and mainly concentrated among those with lower socioeconomic status (uneducated, non-white-collar occupation, and economically disadvantaged). Education is the most commonly considered socioeconomic factor in studies on cognitive impairment, and its protective effect on cognition has been repeatedly demonstrated [18, 43]. Socioeconomic conditions other than good education are also important for older adults’ cognitive health, including white-collar occupations and better economic status [16, 44]. Studies in both developed and developing countries [17, 19] have depicted how an individual’s socioeconomic status affects their cognitive function by the exposure to the resources available and the environment. Better socioeconomic status generally suggests higher quality of life, wider social information, and greater access to health care, which leads to more cognitive stimulation and cognitive reserve to prevent or delay the onset of cognitive impairment [17]. Surprisingly, among the three levels of education in this study, we did not detect a significant protective effect of the highest education level against cognitive impairment. The reason for the insignificance may lie in the confounding caused by the criterion of outcome variable related to education level. Since the sensitivity of MMSE scores to mild cognitive impairment is relatively low [37, 45], there may be some overlap between groups with different MMSE scores [13]. As indicated in previous research, MMSE in the CLHLS may weaken the impact of education [4].

The decomposition results suggested that all proposed variables (except residential area and organized activities) have positive contributions to income-related inequality in cognitive impairment. Demographic factors such as sex, age, and marital status showed a profound impact on older adults’ cognitive impairment. Older women generally have poorer cognitive health and a higher prevalence of cognitive impairment than the older man, which was in line with the findings of previous studies [14, 46]. It is important to realize that women being exposed to gender discrimination since their childhood due to the long-term influence of traditional concepts and cultural norms, they have less access to education and fewer financial resources in comparison with men [47]. Increasing age is a well-known major risk factor of cognitive impairment and dementia [48]. Studies have shown that with the increase of age, the effectiveness of dopamine neurotransmission decreases [49] and different degrees of microvascular dysfunction occur, thus affecting the decline of cognitive function [50]. Moreover, the possibility of cognitive impairment in the old-old is much higher than that in the young-old. Being married and living with a spouse was a significant protective factor against cognitive impairment among older adults, which supports prior research findings [51, 52]. A stable marital relationship can ensure daily care and emotional sustenance for older adults, while those who are separated, divorced, widowed, or never married may suffer from loneliness, insecurity and passive attitude due to a lack of marital support, making them more vulnerable to cognitive impairment [53]. Residence contributed little to the inequality in cognitive impairment among older adults. There was no significant difference in the prevalence of cognitive impairment between urban/town and rural elderly in this study. On the one hand, it is possible that China has promoted equality and industrialization in rural areas in recent decades, which leads to the improvement of infrastructure, Internet construction, and health care systems in rural areas [16]. Rural residents have more access to receive cognitive stimulation than before, and the conditions for prevention and treatment of cognitive decline are more favorable. On the other hand, although urban/town areas can provide a more cognition-stimulating environment than rural area [54], urban/town areas also furnished an adverse environment (such as fragmentation of family structure and support, reduced social interaction) that drives chronic stress and cognitive abnormalities in disadvantaged individuals [55].

Previous research has shown that social support plays a vital part in later life [24] and is associated with overall cognitive performance in older adults [33]. As a psychosocial factor, social support can shape older adults’ positive attitudes and provide a “transition zone” to buffer the negative effects associated with stressful events [56]. In the multivariate analysis, different sources of emotional support play a protective role in cognitive impairment. Studies of elderly Chinese have shown that children are irreplaceable spiritual pillars [24]. Emotional support from their children is one of the important factors affecting their mental health [57], which is related to a reduced risk of cognitive impairment. The emotional support from the spouse again emphasized the importance of marriage for the healthy life of older adults [24]. Besides, connections with friends, neighbors, and others (meaning richer sources of information) also indicated the significant role of support from outside the family for older adults’ cognitive health [58]. In addition, in terms of the resources available to older adults in China, it is common for older adults to receive financial support from family members [25], especially for those in rural areas, because they are less likely to be covered by pensions and health insurance [59]. Access to financial support can help them prevent the insufficient cognitive condition and underutilization of health services resulting from financial dependence. Regarding living arrangement, results showed that older adults living in an institution or with the household member(s) have a higher risk of cognitive impairment than older adults who were living alone, which confirmed the results of previous studies that older adults who live alone are in better health than those who live in a nursing home or with family member(s) [25, 60]. The higher concentration of cognitive impairment among older adults living in an institution or with family member(s) also confirmed that older adults with impairment are more likely to use informal or formal care. The more severe the illness or disability, the formal institutional care is more required [61].

The relationship between social participation and cognitive impairment varies with the type of social participation. Informal activities such as group dancing and singing, playing cards/mahjong are the major forms of activities for Chinese older adults to participate in society [34], and these activities may be of use to delay cognitive decline. Previous studies have suggested that participation in group leisure activities can help older adults relieve negative emotions and gain self-efficacy while remaining physically [62] or cognitively active [63] for direct health benefits. In China, especially for older adults, participation in organized activities is relatively limited due to barriers such as age discrimination, lack of access to information, and a greater emphasis on contact with the family rather than the outside world [34]. In our study, only 14.08% of respondents said they had participated in organized activities. This may provide an explanation for the insignificant effect of participation in organized activities on the prevalence of cognitive impairment.

The findings have some implications for reducing inequalities in cognitive impairment among older adults. Firstly, attention to education should be given to society from childhood, especially to the girl child. The promotion of education enables people to get better jobs and incomes, and the gap between people’s socioeconomic status can be controlled within a relatively reasonable range, thus reducing the inequalities of cognitive impairment. For older adults, establishing colleges for the aged to provide a leisure platform and further stimulate their cognitive function to remain active. Secondly, it is necessary to cultivate a favorable atmosphere of filial piety to play the role of emotional comfort and financial support brought by children in combating cognitive impairment. Moreover, other associated policies and practices should also be in their place. The integration of community resources and improvement of service capacity should be strengthened to carry out comprehensive community projects, providing more effective follow-up services, such as nursing training, for individuals of low socioeconomic status with cognitive impairment and their family caregivers. Besides, the communities can provide a convenient environment for all forms of activities, including but not limited to establish online or offline mutual aid/friendship groups, to facilitate the generalization of social participation among older adults.

This study was also strengthened by some features. A key strength was that this study is the first study using the decomposition methodology to quantify the inequality in cognitive impairment and its influencing factors, it helps to better estimate and explain the degree of inequalities in cognitive impairment and the contributions of determinants. The second strength was that this study provides a comprehensive insight into the social determinants of inequalities in cognitive impairment through combining the previous piecemeal evidence. Another strength was that the measurement of inequality in cognitive impairment was based on a large national sample among Chinese older adults, making the results more convincing and representative. Nevertheless, this study has some limitations. First of all, some variables were not conceptualized and operationalized well, which may impact the validity of the findings. Partly because of the use of self-reported data, some responses may be influenced by social desirability biases. And partly due to the limited data of CLHLS, the education on an upper-secondary and tertiary level have not been explored, the evaluation of emotional support by posing only one question may be not precise enough, the neglect of certain populations (single people, LGBT, couples without children, etc.) can bias the setting of financial support, and never married persons were not separated from separated/divorced/widowed persons. Second, there may be other potential social variables and non-social variables that need to be controlled and need more comprehensive research in the future. For instance, individuals who had been diagnosed with neurodegenerative disease or dementia were not excluded from the CLHLS, which may affect the outcome. Third, the research based on cross-sectional data may not claim causality, which may need to be investigated by establishing panel data in the future.

## Conclusion

This study provides evidence of socioeconomic-related inequalities in cognitive impairment among Chinese older adults disfavoring the poor population. Older adults who are socioeconomically vulnerable require more policy attention, including elderly women, the older elderly, widowed elderly, illiterate elderly, elderly in poor economic conditions, individuals without social support, and individuals who are less involved in social. The findings suggest that socioeconomic-related strategies are beneficial to reduce the inequality in cognitive impairment among older adults. The first strategy is to promote education coverage to reduce accumulated socioeconomic status gaps in old age. The second is that filial piety’s social atmosphere should be encouraged to help older adults prevent cognitive impairment through family members’ support. The third strategy is to improve the care for older adults by developing community care programs and facilities with the community and social workers’ help.

## Data Availability

Publicly available datasets were analyzed in this study. Please contact CLHLS for data requests: http://162.105.138.117/dataset.xhtml?persistentId=doi:10.18170/DVN/WBO7LK.

## Abbreviations

CLHLS: Chinese Longitudinal Health Longevity Survey
MMSE: Mini-Mental State Examination
C: Concentration index
